# Letter to the editor

**DOI:** 10.1080/02813432.2025.2487094

**Published:** 2025-04-08

**Authors:** Håkan Hanberger, Anders Ternhag, Enrico Baraldi, Sofia Wagrell, Charlotta Edlund

**Affiliations:** a Department of Infectious Diseases in Region Östergötland, Linköping University Hospital, Linköping, Sweden; b Department of Biomedical and Clinical Sciences, Linköping University, Linköping, Sweden; c Public Health Agency of Sweden, Solna, Sweden; d Department of Medicine Solna, Karolinska Institute, Stockholm, Sweden; e Department of Business Studies, Uppsala University, Uppsala, Sweden; f Division of Industrial Engineering and Management, Department of Civil and Industrial Engineering, Uppsala University, Uppsala, Sweden

Dear Editor,

We read with interest the paper by Plejdrup Hansen et al. 2024 in the Journal [1] suggesting harmonizing of treatment recommendations for phenoxymethylpenicillin (PcV) against acute respiratory tract infections (ARTIs) in Scandinavia. We welcome this effort to harmonize treatment recommendations for PcV against ARTI, which could reduce the diversity of strengths of tablets and size of packages used in Nordic countries. Whether this would also mean a less costly production and distribution chain, higher profitability and ultimately more stable supply for health care requires a more detailed analysis at country and supplier levels. In the paper the authors refer to a report from our platform for innovation of existing antibiotics [2] showing that PcV tablets are marketed in a total of 10 strengths and in 41 different package sizes in Denmark, Finland, Norway and Sweden. To improve availability of vulnerable generic antibiotics in the Nordic market we have proposed several actions to reduce the risk of shortages. However, we found that there are significant differences between the Nordic countries in price settings systems, procurement, rules for reporting upcoming shortages to national authorities and coordination of measures in case of shortages. Thus, effective actions to reduce shortages may differ amongst the Nordic countries due to contextual differences. However, harmonization of treatment recommendations could be a feasible option regardless of these disparities. Another action that could be a “game changer” for all Nordic countries would be to provide production capacity of essential antibiotics in pharmaceutical industrial plants with infrastructure for producing betalactam antibiotics. A first step could be a limited production with capacity to rapidly scale up in emergent situations of global prolonged shortages that also affect the Nordic countries. A common Nordic production capacity of antibiotics needs to be further investigated in collaboration with the pharmaceutical industries, health care organizations and governments. One way forward could be for the governments to sign preparedness agreements with the pharmaceutical industries for production in crisis/shortages situations. These agreements should include contracted production volumes on call-offs, and defined status regarding regulatory, legal and commercial conditions.

There are many reasons for shortages of generic antibiotics, most of which are strongly related to the low revenues of antibiotics compared to other medicines. Pharmaceutical companies therefore prioritize production and sustainable access of more profitable drugs. Since the Nordic pharmaceutical market is approximately 1% of the European market we need to invest in joint actions that provides sustainable access for all Nordic countries both for short and long term shortages. Efforts as described in the paper by Plejdrup Hansen et al. 2024 (1) to harmonize treatment recommendations for PcV against ARTI, can be used for other indications as well, with the aim to reduce the fragmentation of the Nordic market. Such harmonization may improve the supply situation, but most probably there is a need for coordination of price setting mechanisms and procurement as well to prevent short term shortages. However, to overcome *long term shortages*, the Nordic countries may need to go beyond harmonization initiatives and provide production capacity of essential antibiotics in a Nordic collaboration.

## References

[1] Plejdrup Hansen M, Høye S, Hedin K. Antibiotic treatment recommendations for acute respiratory tract infections in Scandinavian general practices-time for harmonization? Scand J Prim Health Care. 2025;43(1):205–208. doi: 10.1080/02813432.2024.2422441. 39494720 PMC11834794

[2] https://www.platinea.se/download/18.372739df18d8724e32efbba/1707745814389/240212_ Mappingtheantibioticmarket_PLATINEA.pdf

